# Factorial Invariance of the 10-Item Connor-Davidson Resilience Scale Across Gender Among Chinese Elders

**DOI:** 10.3389/fpsyg.2019.01237

**Published:** 2019-05-31

**Authors:** Meng Meng, Jiayue He, Yuzhu Guan, Haofei Zhao, Jinyao Yi, Shuqiao Yao, Lezhi Li

**Affiliations:** ^1^ Department of Nursing, Second Xiangya Hospital, Central South University, Changsha, China; ^2^ Xiangya School of Nursing, Central South University, Changsha, China; ^3^ Medical Psychological Center, Second Xiangya Hospital, Central South University, Changsha, China

**Keywords:** factorial invariance, resilience, aged, factor analysis, reliability

## Abstract

Resilience plays an important role in the health of the elderly. The 10-item Connor-Davidson Resilience Scale (CD-RISC-10) is widely used to evaluate resilience, but its factorial invariance has not been evaluated in the Chinese elders. In the current study, 1,238 Chinese elders aged 60 years and above completed the Chinese CD-RISC-10, yielding good reliability (Cronbach’s *α* = 0.936, Omega coefficient = 0.83, and test-retest reliability coefficient of 0.665 after 6 months). Confirmatory factor analysis indicated that a single-factor model fitted our CD-RISC-10 data well, both for the total sample and for each gender group. Furthermore, factorial invariance across genders was supported by multigroup confirmatory factor analysis. Finally, the current study revealed greater resilience levels in Chinese elderly women than in Chinese elderly men.

## Introduction

Given China’s very large population and the recent sharp increase in the aging population in China, the physical and mental health of the elderly are attracting substantial attention in China. Defined as an individual’s ability to cope with adversity and bounce back from difficult experiences (Campbell-Sills and Stein, 2007), resilience has become an important consideration of geriatric mental health because it is key to enabling elderly persons to overcome adverse psychological problems (Connor and Davidson, 2003; Guo et al., 2015). Resilience, which has been shown to not only help reduce morbidity risk, alleviate loneliness, enhance stress-coping ability, and support the maintenance of cognitive and physical functioning of the elderly, may also relieve depressive symptoms associated with stressful life events (Hildon et al., 2010; Lou and Ng, 2012; Fontes and Neri, 2015; Lim et al., 2015; Niu et al., 2016). Thus, it is of great public health significance to study the resilience of the elderly in China.

The 25-item Connor-Davidson Resilience Scale (CD-RISC), which was developed by Connor and Davidson in 2003 to quantify resilience and assess treatment response, is a widely used clinical tool with very good psychometric ratings (Connor and Davidson, 2003; Windle et al., 2011). However, the factor structure of the CD-RISC differs across countries, living environments, and age bands. In a study of 577 healthy adult American participants, exploratory factor analysis revealed a five-factor CD-RISC structure (personal competence, high standards, and tenacity; trust in one’s instincts, tolerance of negative affect, and strengthening effects of stress; positive acceptance of change and secure relationships; control; and spiritual influences) (Connor and Davidson, 2003). Meanwhile, in a study of 1,395 community-dwelling American women over 60 years of age, a four-factor structure (personal control and goal orientation; adaptation and tolerance for negative affect; leadership and trust in instincts; and spiritual coping) was obtained (Lamond et al., 2009). In a study of 783 Spanish entrepreneurs operating in the business services sector, a three-factor structure (hardiness; resourcefulness; and optimism) was obtained (Manzano-García and Ayala Calvo, 2013). Likewise, in a study of 246 Turkish earthquake survivors, a three-factor structure (tenacity and personal competence; tolerance of negative affect; and tendency toward spirituality) was observed (Karairmak, 2010). A three-factor structure (tenacity; strength; and optimism) was also obtained with the Chinese version of the CD-RISC in a study of 560 Chinese residents of Guangdong and Beijing (Yu and Zhang, 2007).

Given the various factor structures reported for the 25-item CD-RISC, Campbell-Sills and Stein revised the scale in 2007 into a refined 10-item single-dimension CD-RISC (CD-RISC-10). In a cohort of 1,743 undergraduates, exploratory and confirmatory analyses demonstrated good internal reliability (Cronbach’s *α* = 0.85) and construct validity of the CD-RISC-10 (Campbell-Sills and Stein, 2007), indicating that the abridged CD-RISC is a reliable, valid assessment tool, in addition to being easier to apply clinically, relative to the 25-item CD-RISC, owing to its simplicity. The CD-RISC-10 has been translated into several languages, and it has been tested on various populations including Canadian college women, Danish hospital staff, Khmer adolescents, American competitive long-distance runners, French women, Brazilian young people, Spanish nonprofessional caregivers, and low-income African American men, among others (Lopes and Martins, 2011; Scali et al., 2012; Coates et al., 2013; Duong and Hurst, 2016; Gonzalez et al., 2016; Blanco et al., 2017; Lauridsen et al., 2017; Hébert et al., 2018). The Chinese version of the CD-RISC-10 has been reported to be useful for assessing mental resilience quickly in a cohort of Chinese parents of children with cancer (Ye et al., 2017) and was also reported to have good psychometric properties in a study of Wenchuan earthquake survivors (Wang et al., 2010). In addition to having been widely applied, the CD-RISC-10 has also been shown to have good internal consistency, with Cronbach’s *α* values in the range of 0.81–0.95 (Wang et al., 2010; Aloba et al., 2016; Shin et al., 2018).

Some researchers have reported that exposure to trauma in females is associated with a reduced resilience score (Stratta et al., 2013; Hirani et al., 2016). However, due to the lack of data on measurement invariance across genders, we cannot infer the causes of the differences observed because group comparisons require equivalent measurement. To the best of our knowledge, no confirmatory factor analysis study has tested the measurement invariance of the CD-RISC-10 across gender groups in an elderly Chinese cohort.

The current study had four aims. First, we tested the reliability of the CD-RISC-10 in an elderly Chinese study cohort. Second, we examined the model fit of the CD-RISC-10 in a community sample of Chinese elderly. Third, we examined the factorial invariance of the CD-RISC-10 across gender groups. Finally, upon establishment of adequate factorial invariance, we planned to compare resilience scores between men and women.

## Materials and Methods

### Participants and Procedure

This study was conducted in the communities of Beijing, Shandong and Hunan provinces of mainland China. The questionnaires were distributed by well-trained staff to elderly residents aged 60 years and above who came to the community activity center. The staff provided help for participants who had visual impairment, could not read or fill out the questionnaire themselves. The inclusion criteria of this study were: 60 years old and above; agree to participate in this study. The exclusion criteria included: diagnosed with severe mental illness; insufficient cognitive ability to understand the questionnaire; unable to understand mandarin and therefore unable to complete the questionnaire; cannot fill out the questionnaire due to other reasons. A total of 1,284 participants returned questionnaires, but 46 failed to respond to all 10 items. Thus, the final sample included 1,238 (96.4% completion rate). The mean age of the final sample was 71.64 years [standard deviation (SD) = 7.77]. The final sample consisted of 525 men (42%), with a mean age of 72.47 years (SD = 8.09) and 713 women (58%) with a mean age of 71.02 years (SD = 7.46). The study was approved by the ethics committee of Second Xiangya Hospital, Central South University. All participants provided written informed consent at the time of enrollment.

### Instrument

The CD-RISC-10, which consists of 10 items, was derived from the original 25-item CD-RISC. It assesses an individual’s mental resilience during the past month, such as “Adapt to change” (see the items in the Appendix). Respondents rate each item on a 5-point Likert scale from 0 (not true at all) to 4 (true nearly all the time). The item ratings are summed to produce a scale score ranging from 0 to 40, with higher values implying a greater resilience capability. The Chinese version of the CD-RISC-10 employed in this study has been confirmed to have good internal consistency (Cronbach’s *α* = 0.851–0.910) and excellent structure validity in Chinese populations (Wang et al., 2010; Ye et al., 2016, 2017).

### Data Analysis

Mean values are reported with standard deviations (SDs). Data management was carried out in SPSS 18.0 and confirmatory factor analysis was conducted in Mplus 6.11. Kolmogorov-Smirnov normality testing on item scores showed significant deviation from the normal distribution (all *p* < 0.001, see Table 1) indicating that the data were not normally distributed. Based on the above, the robust maximum likelihood estimator was chosen for data analysis because it, when applied with a mean-adjusted Chi-square (Satorra-Bentler *χ^2^*) statistic and robust standard errors, yields an unbiased goodness-of-fit index that is robust to nonparametric data (Satorra and Bentler, 2001; Wang et al., 2013). The data analysis was conducted in three steps, as delineated below:

**Table 1:** Descriptive statistics for each item of the Chinese CD-RISC-10.

Item	Mean	SD	Item factor loading	Skewness	Kurtosis	Kolmogorov-Smirnov *Z*	*p*
1	2.81	0.99	0.75	−0.74	0.27	8.82	0.000
2	2.92	0.93	0.82	−0.73	0.35	8.64	0.000
3	2.52	1.04	0.74	−0.40	−0.40	7.71	0.000
4	2.93	0.92	0.84	−0.78	0.53	8.93	0.000
5	2.87	0.92	0.80	−0.80	0.63	9.53	0.000
6	2.76	0.96	0.84	−0.79	0.51	9.76	0.000
7	2.76	0.95	0.84	−0.67	0.24	9.47	0.000
8	2.78	1.06	0.73	−0.91	0.39	9.83	0.000
9	2.99	0.94	0.84	−0.93	0.73	8.97	0.000
10	2.87	0.95	0.81	−0.83	0.59	9.36	0.000

Note: SD, Standard deviation.

In the first step, reliability analysis was conducted. We used Cronbach’s *α* value, McDonald’s Omega coefficient, and test-retest reliability coefficient to determine the reliability of the CD-RISC-10.

In the second step, we used confirmatory factor analysis to test the goodness of fit of the single factor structure of the Chinese CD-RISC-10 in the total sample and each gender group. Chi-square (*χ^2^*) and standardized root mean squared residual (SRMR) tests were employed as absolute fit indexes. Because the *χ^2^* test can be affected by sample size, especially in large samples, we also applied the root mean square error of approximation (RMSEA) as parsimony fit index and applied the Comparative Fit Index (CFI) and Tucker-Lewis Index (TLI) as comparative indexes. The following previously established criteria of acceptability were used: SRMR ≤0.08, RMSEA ≤0.08, CFI ≥ 0.90, and TLI ≥ 0.90 (Hu and Bentler, 1999; Brown, 2006; He et al., 2019).

In the third step, multigroup confirmatory factor analysis was undertaken to evaluate the factorial invariance of the CD-RISC-10 across gender groups. The invariance tests were completed for configural invariance (Model 1), metric invariance (Model 2), scalar invariance (Model 3), strict invariance (Model 4), factor variance/covariance invariance (Model 5), and factor latent mean invariance (Model 6) (He et al., 2018). First, we conducted configural invariance tests (without parameter constraints) to evaluate the latent variable structure across gender groups, the results of which served as a baseline model for subsequent tests. Then, metric invariance was tested based on the configural invariance results with factor loading equivalence constraints imposed to ensure similarity of the observed indicators and underlying traits across gender groups. Next, we applied a scalar invariance test in which we constrained both factor loadings and intercepts of variables equally across genders to test for an intergroup difference in the measured intercept based on the result of last step. Subsequently, strict invariance testing was conducted with factor loading, variable intercepts, error variance constraints equally set. Following the measurement equivalence testing, factor variance/covariance invariance and factor latent mean invariance tests were conducted to evaluate the structural invariance of the Chinese CD-RISC-10. We employed the Bayesian information criterion (BIC) and TLI and CFI changes to evaluate invariance across consecutive models. In accordance with published recommendations (Raftery, 1995; Cheung and Rensvold, 2002; Wu et al., 2012; Xiao et al., 2014), a ΔTLI ≤0.010 and a ΔCFI ≤0.010 with a smaller BIC value were considered evidence of invariance. Finally, a nonparametric test, Mann-Whitney U test, was used to compare CD-RISC-10 scores across the gender groups. Because the Kolmogorov-Smirnov normality test showed that the scores of two samples do not conform to the normal distribution, and therefore we conservatively considered that whether the scores of CD-RISC-10 in Chinese elderly men and women conform to the normal distribution remained uncertain.

## Results

### Descriptive Data and Analyses of Reliability of the 10-Item Connor-Davidson Resilience Scale

Descriptive statistics, including mean scores with SDs, the skewness, and the kurtosis, for each item of the CD-RISC-10 are reported in Table 1. The mean scores (SDs) for item 1 through 10 were 2.81 (0.99), 2.92 (0.93), 2.52 (1.04), 2.93 (0.92), 2.87 (0.92), 2.76 (0.96), 2.76 (0.95), 2.78 (1.06), 2.99 (0.94), and 2.87 (0.95). And the skewness values were −0.74, −0.73, −0.40, −0.78, −0.80, −0.79, −0.67, −0.91, −0.93, and − 0.83 for item 1 to 10 while the kurtosis values were 0.27, 0.35, −0.40, 0.53, 0.63, 0.51, 0.24, 0.39, 0.73, and 0.59. According to the skewness and kurtosis values of each item, it can be seen that the mean score of each item presented a negative skewness distribution, and the kurtosis value was close to 0. Overall, the mean (SD) total CD-RISC-10 scores were 27.60 (8.09) for males and 28.68 (7.39) for females. In our study, the Cronbach’s *α* of the CD-RISC-10 was 0.936, the McDonald’s Omega coefficient was 0.83, and the test-retest reliability coefficient was 0.665 after 6 months (*N* = 124).

### Confirmatory Factor Analysis

As reported in Table 2, we obtained a good fit index for the full sample, the male group, and the female group. Briefly, all TLI, CFI, RMSEA, and SRMR values were > 0.90, >0.90, <0.08, and < 0.08, respectively, indicating that the single-factor model fit the data well in the total sample and each gender group. These results confirmed that the single-factor model can be used as a baseline model for subsequent tests.

**Table 2:** Goodness of fit indexes for the CD-RISC-10 model.

Group	S-B*χ^2^*	*df*	TLI	CFI	RMSEA	SRMR
Full sample (*N* = 1,238)	233.195	35	0.949	0.961	0.068	0.031
Males (*N* = 525)	117.143	35	0.952	0.963	0.067	0.032
Females (*N* = 713)	145.940	35	0.950	0.961	0.067	0.033

Note: S-Bχ^2^, Satorra-Bentler scaled χ^2^; df, Degrees of freedom; TLI, Tucker-Lewis Index; CFI, Comparative fit index; RMSEA, Root mean square error of approximation; SRMR, Standardized root mean squared residual.

### Factorial Invariance

The factorial invariance test results, including Satorra-Bentler scaled *χ^2^* values with degrees of freedom, TLI values and inter-model differences, CFI values and inter-model differences, and BIC values are reported in Table 3. The fit indexes of each successive model from Model 1 to Model 4 met the satisfactory fit criteria. That is, between successive models (1 to 2, 2 to 3, and 3 to 4), the ∆TLIs were all <0.010 and the ∆CFIs were all <0.010. The successive decreases in BIC values were 57.891 from Model 1 to Model 2, 53.561 from Model 2 to Model 3, and 60.839 from Model 3 to Model 4. Because these four steps of measurement invariance were performed in sequence, we drew the conclusion that the assumption of measurement invariance across gender was established.

**Table 3:** Fit indices for invariance tests of the CD-RISC-10.

Model	S-Bχ^2^	*df*	TLI	CFI	RMSEA (90%CI)	SRMR	Comparison	∆TLI	∆CFI	BIC
1	261.409	70	0.951	0.962	0.066 (0.058–0.075)	0.033	–	–	–	26484.037
2	276.489	79	0.955	0.961	0.064 (0.055–0.072)	0.036	2 vs. 1	0.004	−0.001	26426.146
3	295.703	88	0.958	0.959	0.062 (0.054–0.070)	0.036	3 vs. 2	0.003	−0.002	26372.585
4	286.384	98	0.965	0.962	0.056 (0.048–0.063)	0.037	4 vs. 3	0.007	0.003	26311.746
5	289.818	99	0.965	0.962	0.056 (0.048–0.063)	0.064	5 vs. 4	0.000	0.000	26309.930
6	294.466	100	0.965	0.961	0.056 (0.049–0.064)	0.068	6 vs. 5	0.000	−0.001	26308.937

Note: Model 1, Configural invariance; Model 2, Metric invariance; Model 3, Scalar invariance; Model 4, Strict invariance; Model 5, Factor variance/covariances invariance; Model 6, Factor latent mean invariance; S-Bχ^2^, Satorra-Bentler scaled χ^2^; df, Degrees of freedom; TLI, Tucker-Lewis Index; CFI, Comparative fit index; RMSEA, Root mean square error of approximation; CI, Confidence interval; SRMR, Standardized root mean squared residual; BIC, Bayesian information criterion.

The TLI and CFI values were unchanged from Model 4 to Model 5 (variance/covariance equivalent), with only a 1.816 decrease in BIC. Similarly, from Model 5 to Model 6 (factor latent mean invariance), there was no change in TLI and only a negligible change in CFI, with a BIC decrease of only 0.993. Hence, the ∆TLI and ∆CFI were < 0.010 in both comparisons, with BIC values smaller than in the factor variance/covariance equivalent model. Therefore, we concluded that the factorial invariance across gender among Chinese elders was established.

### Gender Difference

The female group had a higher total CD-RISC-10 score, at 28.68, than the male group, at 27.60 (*Z* = −2.373, *p* = 0.018). On items 4, 6, 8, and 9, the female group had significantly higher scores than the male group (all *p <* 0.05). On items 1, 2, 3, 5, 7, and 10, there was no significant difference in scores between the two groups (all *p* > 0.05). The mean rank and *p* of each item and the comparison between the two gender groups are given in Table 4.

**Table 4:** Comparisons of each item of CD-RISC-10 between male and female.

Item	Mean rank		*Z*	*p*
	Male	Female		
1	601.39	632.84	−1.164	0.107
2	602.40	632.09	−1.530	0.126
3	601.00	633.12	−1.630	0.103
4	584.97	644.92	−3.101	0.002
5	609.71	626.71	−0.884	0.377
6	597.36	635.81	−1.998	0.046
7	605.59	629.74	−1.251	0.211
8	595.72	637.01	−2.128	0.033
9	585.12	644.81	−3.089	0.002
10	609.37	626.96	−0.910	0.363
Total score	591.44	640.16	−2.373	0.018

## Discussion

The main aim of our study was to probe the psychometric properties of the Chinese version of the CD-RISC-10 in an elderly Chinese population. The Cronbach’s *α* value and the test-retest reliability coefficient indicated that the single-factor Chinese CD-RISC-10 has good internal consistency. The present findings indicate that the CD-RISC-10 is a stable and consistent measurement.

Subsequent multiple group confirmatory analysis performed to estimate the measurement equivalence of the scale across genders showed that the model fitted well in the full sample and in each gender group. Importantly, the results supported configural invariance, metric invariance, scalar invariance, strict invariance, factor variance/covariance invariance, and factor latent mean invariance across genders, confirming full equivalence of the scale across genders. Configural invariance indicates that the pattern of fixed and free parameters was equivalent across genders, with a similar psychological structure being reflected by the same variables in men and women. Subsequent establishment of metric invariance revealed that the relative factor loadings of the items were also equivalent between the two gender groups, indicating that individuals with the same scores on latent variables also scored equally on observation items. In terms of achieving scalar invariance, it was demonstrated that the observed variable intercepts and CD-RISC-10 reference points were the same for men and women. The attainment of strict invariance suggests that differences in latent variable variation could reflect the observed variable variation differences of the scale. Factor variance/covariance invariance and factor latent mean invariance (a.k.a. structural invariance) were established in the current study, indicating that the observed variables and latent variables possessed the same relationship across the two groups. Consequently, we have concluded that the Chinese version of the CD-RISC-10 estimates latent resilience equivalently across genders and thus can be used to compare mental resilience between elderly men and women in China.

The present finding of a significantly higher CD-RISC-10 total score in women than in men suggests that elderly Chinese women may be generally more resilient than elderly Chinese men, consistent with a previous study in China (Lei et al., 2008). However, in other countries, some studies have reported higher resilience scores for men than women (Stratta et al., 2013). It has been hypothesized that males may be better adapted to traumatic events than women, thus resulting in a “protective model” of resilience (Luthar and Zelazo, 2003). This inconsistency between findings obtained in China and findings obtained elsewhere may be due to social and cultural differences. In China, women are encouraged to seek help, which may yield a stronger social support system for dealing with the pressures of life (Lei et al., 2008). Our findings suggest that elderly women in China may be able to deal with negative emotions, such as stress, more easily and with a faster stress recovery than men in China.

## Conclusion

The results of this study indicate that the Chinese version of the CD-RISC-10 has good reliability and meets resilience measurement standards well when administered to both elderly men and elderly women living in Chinese communities. Thus, it can be applied as a reliable tool for testing mental resilience and performing inter-gender comparisons of mental resilience. To the best of our knowledge, this study was the first study to assess the factorial invariance of the CD-RISC-10 in elderly Chinese men and women. Our findings confirmed that factorial invariance of the CD-RISC-10 had been established across gender among Chinese elders. Finally, the present results provide evidence of Chinese elderly women having better mental resilience than Chinese elderly men, and further demonstrated that this gender difference could not be attributed to a gender-dependent scale variance, but rather reflect a true gender difference.

## Data Availability

The raw data supporting the conclusions of this manuscript will be made available by the authors, without undue reservation, to any qualified researcher.

## Author Contributions

SY conceived and designed the study. LL and JY supervised the study. MM performed the analysis and wrote paper. JH contributed to the analysis. YG and HZ collected the data. All co-authors revised and approved the version to be published.

### Conflict of Interest Statement

The authors declare that the research was conducted in the absence of any commercial or financial relationships that could be construed as a potential conflict of interest.

## Appendix

Items of the CD-RISC-10 (English version)

1. Adapt to change
2. Deal with whatever comes my way
3. See humorous side of things
4. Stress makes me stronger
5. Bounce back after illness or injury
6. Believe I can achieve goals despite obstacles
7. Under pressure I stay focused
8. Not easily discouraged by failure
9. Think of myself as a strong person when facing challenges
10. Able to handle unpleasant feelings
